# Effectiveness of interactive augmented reality-based telerehabilitation in patients with adhesive capsulitis: protocol for a multi-center randomized controlled trial

**DOI:** 10.1186/s12891-021-04261-1

**Published:** 2021-04-26

**Authors:** Seung Mi Yeo, Ji Young Lim, Jong Geol Do, Jae-Young Lim, Jong In Lee, Ji Hye Hwang

**Affiliations:** 1 Department of Physical and Rehabilitation Medicine, Samsung Medical Center, Sungkyunkwan University School of Medicine, Seoul, Republic of Korea; 2 Department of Physical Therapy, General School of Medical Sciences, Konyang University, Daejeon, Republic of Korea; 3 Department of Rehabilitation Medicine, Seoul National University College of Medicine, Seoul National University Bundang Hospital, Seongnam-si, Gyeonggi-do Republic of Korea; 4 Department of Rehabilitation Medicine, Seoul St. Mary’s Hospital, College of Medicine, The Catholic University of Korea, 222, Banpo-daero, Seocho-gu, Seoul, 06591 Republic of Korea

**Keywords:** Adhesive capsulitis, Telerehabilitation, Multi-center randomized controlled trial, Augmented reality

## Abstract

**Background:**

As the primary treatment for adhesive capsulitis, intensive and accurate home exercise is as important as physical therapy in hospitals. Augmented reality (AR)-based telerehabilitation has been implemented recently in various musculoskeletal conditions to increase patient compliance and enable patients to exercise with the correct posture. The objective of this study is to present a protocol for investigating the additive effect of interactive AR-based telerehabilitation in comparison with the usual care for patients with adhesive capsulitis.

**Methods:**

This study presents the protocol of a prospective, multi-center, single-blinded, two-armed randomized controlled trial (RCT). One hundred patients with stage I or II adhesive capsulitis will be recruited at the physical medicine and rehabilitation clinic. Patients will be randomly divided into two groups with 1:1 allocation. The intervention group will receive 3 months of hospital-based physical therapy in conjunction with home-based telerehabilitation. The control group will receive 3 months of hospital-based physical therapy in conjunction with a home-based exercise described in a brochure provided by the hospital. The primary outcome will be the change in passive range of motion (ROM) of the affected shoulder joint from baseline to 12 weeks after baseline assessment. The secondary outcomes will be active ROM, pain measured with the numeric rating scale, shoulder pain and disability index, 36-Item Short Form Survey, EuroQoL-5D-5L, and Canadian Occupational Performance Measure.

**Discussion:**

This will be the first RCT study protocol to investigate the effect of telerehabilitation in patients with adhesive capsulitis. The result of this RCT will determine whether AR-based telerehabilitation is more effective than a brochure-based home exercise program and will provide evidence of the usefulness of “telerehabilitation” using hardware (IoT) and software (monitoring platform) technologies to develop “digital therapeutics” for the future.

**Trial registration:**

This trial was retrospectively registered at the Clinicaltrials.gov website on 20 March 2020, with the identifier NCT04316130.

**Supplementary Information:**

The online version contains supplementary material available at 10.1186/s12891-021-04261-1.

## Background

Adhesive capsulitis, also known as “frozen shoulder,” is a common shoulder disease that clinically presents with pain, stiffness, and dysfunction and is pathologically characterized by inflammation and contracture of the glenohumeral capsule [1]. Although there are differences in the literature, adhesive capsulitis has a high prevalence rate of 2–5% in the general population, with the highest prevalence observed among women between 40 and 60 years of age [2]. Adhesive capsulitis consists of the first “freezing (painful)” stage with increased pain and stiffness for several months, a second “frozen (stiff)” stage resulting in loss of range of motion (ROM) of the shoulder, and a third “thawing (recovery)” stage, in which pain and ROM recover [3].

Adhesive capsulitis is generally described as a self-limiting disorder that without supervised treatment, often recovers within 2–3 years [4]; however, its natural history is not fully known, and recent studies have shown that it may result in long-term disability [1, 4–7]. One systematic review showed that appropriate interventions were essential in reducing long-term disabilities [8]. Rehabilitation including joint mobilization and therapeutic exercise is one of the most important interventions required for adhesive capsulitis [9]. Home exercise programs are as essential as physical therapy in hospital because of cost effectiveness and limited medical resources. Tanaka et al. [10] showed that better compliance with a home exercise program resulted in more improvement in adhesive capsulitis. However, home exercise programs are not easily quantified, and it takes time for doctors to explain and modify postures for each patient. Moreover, the accuracy and frequency of home-based rehabilitation may depend on the patient’s statement, rather than on objective and analyzed data. A key success factor of home-based programs is to increase adherence to the program, for instance through telerehabilitation.

Recently, research on telerehabilitation that uses telecommunication technology to provide rehabilitation services at a distance is increasing [11]. Recent systematic reviews have shown that telerehabilitation was comparable to the standard practice in improving physical function and reducing pain of musculoskeletal disorders, mainly for spine-related or postoperative conditions [12]. Developing and applying an AR-based telerehabilitation with specialized step-by-step exercises for patients with adhesive capsulitis is thought to increase clinical effectiveness and improve patient compliance.

We aim to present a protocol for investigating clinical effects of interactive augmented reality (AR)-based telerehabilitation in addition to hospital-based physical therapy through a randomized controlled trial (RCT) designed for patients with adhesive capsulitis. We hypothesize that interactive AR-based telerehabilitation in conjunction with hospital-based physical therapy would lead to better results than a brochure-based home exercise program with hospital-based physical therapy for 3 months post diagnosis.

## Methods/design

### Study design

**Fig. 1:**
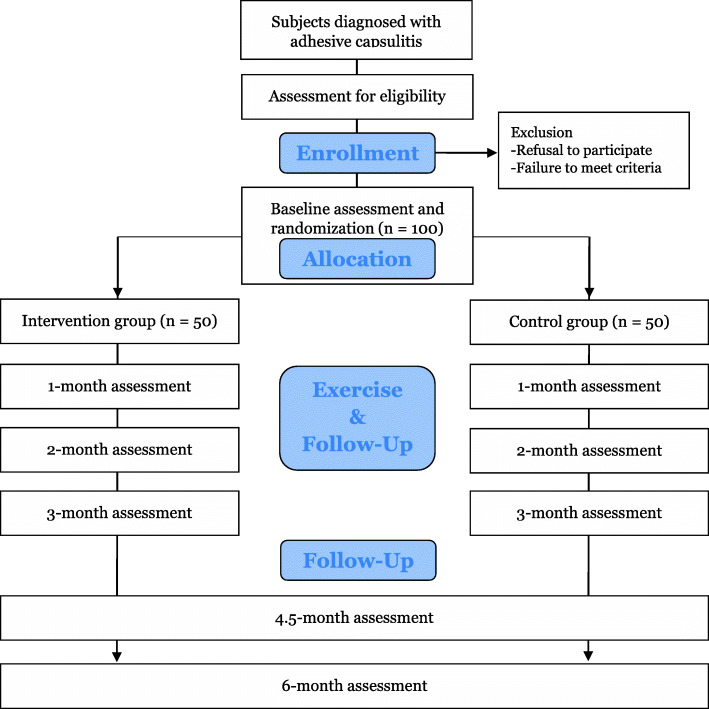
RCT study flow chart

**Fig. 2:**
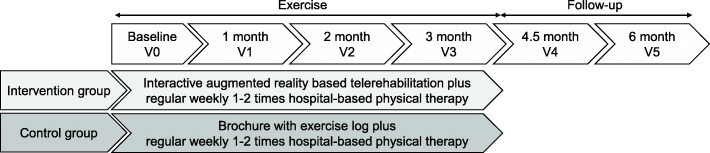
Exercise intervention according to group allocation

This protocol will be a prospective multi-centered single-blinded two-armed RCT. The study design meets SPIRIT guidelines [13] and the SPIRIT Checklist are attached as Additional file 1. All eligible patients will be informed about this study and invited to participate by the outpatient doctor at the Physical Medicine and Rehabilitation clinic in Republic of Korea. All patients submit informed consent if they agree to participate in the study and principal investigator obtained the informed consent. After randomly divided into two groups with a 1:1 allocation, participants will be assessed in baseline. The intervention group will receive at least 3 months of hospital-based physical therapy in conjunction with home-based telerehabilitation. The control group will receive at least 3 months of hospital-based physical therapy in conjunction with a home-based exercise brochure provided by the hospital. The adhesive capsulitis stage will be evaluated at each follow-up by a physician, and exercises according to the stage will be prescribed. Outcomes will be assessed at the baseline and 1-month, 2-month, 3-month, 4.5-month, and 6-month follow-ups. Deviation of 7–15 days from each evaluation point will be allowed. Outcome measures will be performed by the same assessors, who will be blinded to the group allocation. The flow diagram of the study protocol is shown in Figs. 1 and 2.

### Participants

In the protocol, we will enroll 100 participants with stage I or stage II adhesive capsulitis. The inclusion criteria will be as follows:

- Age 19 years or above
- Affected shoulder joint restriction of at least 30° over the contralateral side for two or more of either forward flexion, abduction, or external rotation (with 90° shoulder abduction) when measured by a goniometer in the supine position [13].
- Able to receive in-hospital physical therapy
- Able to install telerehabilitation equipment at home

The exclusion criteria will be as follows:

- Diagnosis of bilateral adhesive capsulitis [14]
- Secondary adhesive capsulitis caused by trauma (shoulder fracture, dislocation) and/or systemic inflammatory disease (rheumatoid arthritis) [13, 14]
- Unable to perform exercise due to general deconditioning
- Communication difficulties

### Randomization, allocation, and blinding

To randomly assign intervention, one researcher will generate a size 4 block randomization list through a computer-generated sequence. To conceal the random allocation, numbered, sealed, and opaque envelopes containing simple explanatory notes will be used to allocate treatment. Independent researcher will allocate the participants using computer-generated sequences with block size of four. The research member involving in randomization process will not participate in the enrollment and assessment process. Participants will be instructed not to tell the assessor which group they are randomized to during exercise and follow-up measurements.

### Interventions

Regardless of group allocation, both groups will receive physical therapy at the hospital once or twice at least for 3 months. Hospital-based physical therapy will be performed for 1 h in combination with joint mobilization and modality (heat and electrical stimulation by physical therapists with more than 10 years of treatment experience). The therapist will be blinded to the assigned intervention group of the participant.

All patients will be instructed to exercise at home at least once a day with precautions for each exercise stage, exercise posture, method, and frequency. Depending on group allocation, there will only be a difference in the way home exercise is performed, with no difference in the program itself or the type/duration of the exercise. All patients will receive an assistant bar, a yellow elastic band and 0.5-kg dumbbells required to perform the exercises. Exercise will consist of 3 stages according to the adhesive capsulitis stage (i.e., stage I is freezing, stage II is frozen, or stage III is thawing). Every month during the intervention period, the exercise stage will change according to the stage of the adhesive capsulitis. All exercises will consist of a warm-up, scapular stabilization, shoulder ROM exercises, and shoulder stretching. The details of the exercise differ by step (Additional file 2), and strength training is added from the frozen stage (stage 2). The stepwise exercise will be the same for both groups and consist of 11–16 types of exercise, ten times per set, with a total exercise time of 30–40 min. Exercise contents of each stage were determined based on the literature [14, 15].

### Telerehabilitation

**Fig. 3:**
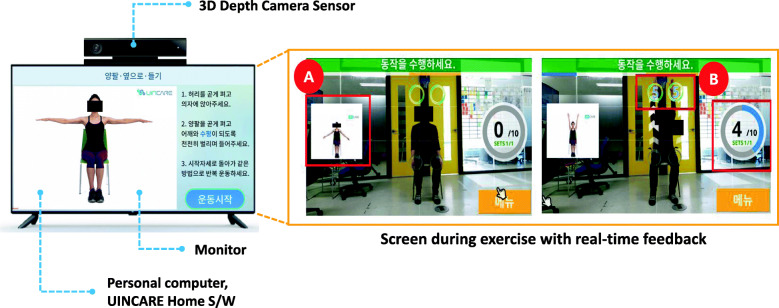
UINCARE Home+ (left) and screen during exercise with real-time feedback (right) (A: guide video, B: holding time, exercise set and number)

For the telerehabilitation, an UINCARE Home+ (UINCARE corp., Seoul, Korea) will be utilized. The UINCARE Home+ will be rented for 3 months and installed at the patient’s home. The main function of this hardware is to capture users’ 25 joint movements in real time through infra-red and motion sensors of 3-dimension depth camera (Xbox One Kinect for Windows®, Microsoft, USA). Exercise will be carried out by the patient viewing and following guide videos. Users can see their movement in the center of the screen as well as the exercise number, holding time, and a small exercise video on the side of the screen. If the user completes the exercise session, they can obtain real-time feedback on performance and accuracy on the result screen (Fig. 3).

**Fig. 4:**
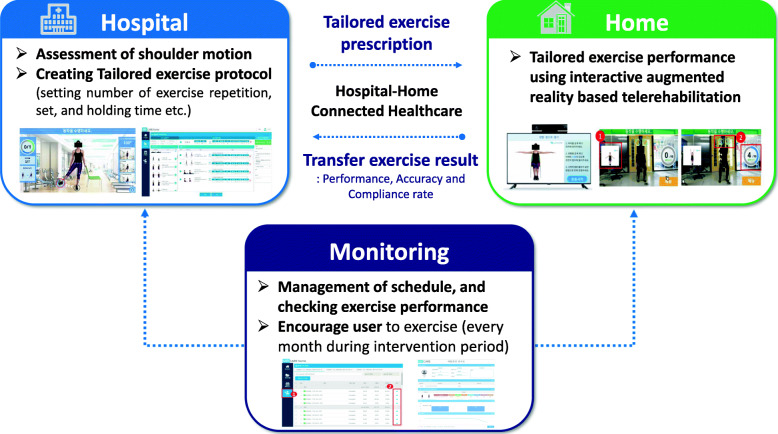
Protocol for interactive augmented reality-based telerehabilitation

The telerehabilitation protocol using the UINCARE Home+ is shown in Fig. 4. First, the physician will diagnose the patient’s stage of adhesive capsulitis and prescribe a corresponding exercise program. The UINCARE company device manager creates the user account and then, independent researchers set up the prescribed exercise program on the administrator page. The UINCARE Home+ will be delivered by the company, installed at the patient’s home, and the patient will be trained on how to use it. During the intervention period, independent researchers will monitor performance, accuracy, and check individual exercise schedules through the administrator page. Every month, the researcher will enquire of the related problem and encourage the patient to exercise at least once a day.

### Brochure (usual care)

This group will be offered a brochure in accordance with the patient’s diagnosed adhesive capsulitis stage. They will be asked to record the number of exercises in their exercise log book. During the intervention period, participants were asked to submit the exercise log book every month.

### Outcomes

Outcome measures that will be assessed by a blinded physical or occupational therapist and time points are described in Table 1.

**Table 1:**
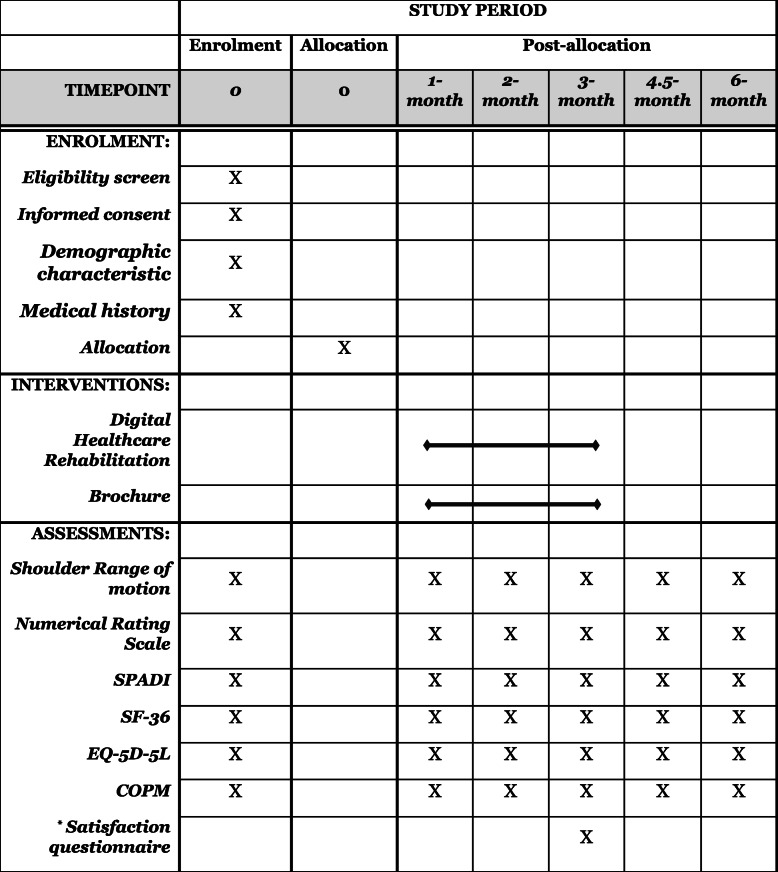
Summary of baseline screening, assessment, and follow-up during study visits

*SPADI* Shoulder pain and disability index, *SF-36* 36-Item Short Form Survey, *EQ-5D-5L* European Quality of Life Five Dimensions Five Level Scale, *COPM* Canadian Occupational Performance Measure

*Digital healthcare system rehabilitation group only

### Primary outcomes

The primary objective of this protocol is to determine the additive effect of interactive AR-based telerehabilitation in conjunction with hospital-based physical therapy on passive range of motion (PROM) for patients with adhesive capsulitis 3 months post diagnosis. Therefore, the primary outcome will be the change in PROM of the affected shoulder joint from baseline to 12 weeks after baseline assessment.

### Secondary outcomes

The secondary objectives of this protocol are to determine the additive effect of interactive AR-based telerehabilitation in conjunction with hospital-based physical therapy on 1) pain intensity; 2) activities of daily living; 3) quality of life; and 4) shoulder disability over time. Therefore, the secondary outcomes will be active ROM, pain measured with the numeric rating scale, shoulder pain and disability index, 36-Item Short Form Survey, European Quality of Life Five Dimensions Five Level Scale, and Canadian Occupational Performance Measure. Along with the RCT, a satisfaction questionnaire (Additional file 3) and safety assessment will be conducted for both the system and equipment.

As secondary outcomes, active range of motion (AROM), pain and disability of the affected shoulder joint, quality of life, and activities of daily living (ADL) will be considered at all follow-up assessments. The satisfaction questionnaire for interactive AR-based rehabilitation will be evaluated only in the intervention group after 12 weeks.

Supine measurements of PROM and AROM of the affected shoulder will be obtained with the participants lying in a supine position. Shoulder forward flexion, abduction, and external and internal rotation with a 90° shoulder abduction will be measured with an electronic goniometer in units of 1°. All ROMs of each participant will be assessed as described by Kelly et al. [14].

The pain intensity at resting and activity will be evaluated by a 11-point Numeric Rating Scale (NRS) [16] for the past week, with 0 and 10 representing “no pain” and “the worst possible pain”, respectively Participants will be asked for an average perceived pain intensity in ADL.

Shoulder pain and disability for the past week will be assessed using the Shoulder Pain and Disability Index (SPADI), consisting of two subscales with a total of 13 items (five items for pain and eight for disability) [17]. Patients will report a level of difficulty performing ADL due to pain and limit of motion. The final score will range from 0 to 130 with a percentage score of 0 indicating less shoulder disability and 100 indicating more shoulder dysfunction.

Quality of life will be assessed using the 36-Item Short Form Survey (SF-36) and the European Quality of Life Five Dimensions Five Level Scale (EQ-5D-5L). The SF-36 consists of eight scaled scores as a patient-reported survey of overall health. Scores for each domain range from 0 to 100, with a higher score defining a more favorable health status [18]. The EQ-5D-5L measures the health-related quality of life of patients. A validated model for the Korean population may be used to interpolate quality weights for all EQ-5D-5L health states [19]. This scale is numbered from − 0.066 to 0.904. Utility will be applied to the economic evaluation of both the intervention and control groups.

The ADL assessment will be used by the Canadian Occupational Performance Measure, which compares the importance, performance, and satisfaction of activities that present problems in daily life and can identify the five most problematic activities before and after intervention. Performance and satisfaction can be obtained by summing the scores of all items and then dividing it by the number of items. If the re-evaluation value minus the pre-evaluation value is 2 or more, intervention is significant [20].

### Sample size calculation

The target sample size was calculated through consultation with an in-hospital statistics team. The primary hypothesis was that there would be a significant difference in the amount of change in PROM before and after 3 months for the digital health care and control groups. The ROM consists of flexion, abduction, external rotation, and internal rotation of the shoulder joint. When the standard deviation of the variation is 18.64, 16.79, 11.22, and 11.65, respectively, and the difference between the two groups variation is expected to be 15, 15, 10, 10, or more, respectively, based on the results of the existing studies [21–25], 35 people per group are required for a significance level of 5% (a significant level of 0.05/4*100% of each hypothesis) and 80% statistical power. Assuming an expected dropout rate of 25%, we require at least 47 participants per group.

### Data management

All participant data will be collected and coded by research team members and will be stored in the secured platform accessible only research team members. Backup database will be updated regularly. The anonymized dataset will be available on request to the corresponding authors. No interim analysis will be performed prior to the end of the study.

### Statistical analysis

Subject demographics will be calculated using descriptive statistics. Kolmogorov–Smirnov test will be performed to assess the distribution of data. To compare baselines between the two groups, an independent t-test and chi-square test will be conducted. To compare the mean difference, if primary outcome (PROM) data in both groups is found to be normally distributed, we will perform an independent t-test, not Mann-Whitney test. How to handle missing data will be applied depending on distribution of data after study completed.

To investigate the effects of the interventions and differences between groups for secondary outcomes, a mixed effects model or generalized estimating equations will be conducted, with one between-subject factor (group: intervention and control) and one within-subject factor (evaluation time: baseline, 4-week, 8-week, 12-week, 18-weeks, and 24-week follow-ups). All data will be analyzed using IBM SPSS Statistics version 20.0 (Armonk, New York, USA) and a significance level of 5% will be set with a 95% confidence interval.

### Participant safety and withdrawal

The potential risk level reviewed by institutional review board and principal investigator is minimal. For prevention and management in adverse events, participants can call researchers at any time if they have any question or problems. If there are an intolerable muscle pain or injury, they will visit a hospital and will be examined their condition by principal investigator.

All participants are able to discontinue voluntarily the study at any time and they can be withdrawn in case of the significant disease non-related to study, and not following instruction of doctor in charge.

### Ethics and dissemination

All study procedures were approved by the Institute Review Board of two hospitals (approval numbers: SMC-2019-05-021 and KC20ENDE0254). The trial was registered on clinical trials.gov (approval ID: NCT04316130). The study protocol was fires reviewed by the institutional review board of Samsung Medical Center on 17 May 2019 and was approved on 7 June 2019 as original protocol. If there are important protocol modifications, principal investigator will share them with coordinating investigators and trial participants, and report to institutional review board.

Personal information about enrolled participants will be collected, shared with clinic (i.e., Seoul St. Mary’s Hospital), and retained by only research team during study. After the end of the study, all personal information will be retained for 3 years and then destroyed.

The trial results will be published in the journal and report of results will be posted on the funding institute’s site for the public, participants, and healthcare professionals.

## Discussion

This study aims to present a protocol for investigating clinical effects of interactive AR-based telerehabilitation in addition to hospital-based physical therapy through a randomized controlled trial designed for patients with adhesive capsulitis. To our knowledge, this RCT would be the first study investigating the effect of telerehabilitation using an IoT device in patients with adhesive capsulitis. As the completion rate and accuracy of using kinetic motion sensors are integrated with a monitoring platform, patients can correctly perform daily home-based shoulder exercises under the remote supervision of a physician.

Adhesive capsulitis is a disorder that requires intensive hospital-home connected treatment with correct posture during a limited time period and requires different exercises depending on the stage and irritability [26]. In other words, adhesive capsulitis is a disease that can be clinically effective in applying telerehabilitation with specifically prescribed exercise programs. To the best of our knowledge, only two other studies have applied telerehabilitation with wearable sensors to patients with adhesive capsulitis. Both studies are similar to this study in that they compared a motion sensor-assisted telerehabilitation group with a classic home exercise group; however, they had a small sample size and were not RCTs [27, 28]. This RCT will use a more advanced technology to improve the quantity (patient compliance) and quality (real-time feedback through AR and telecommunication with physicians) of home exercise.

With physical therapy in hospitals being limited and not currently preferred due to the recent COVID-19 pandemic, the importance of home exercise is more relevant than ever before. However, compliance is poor for home exercise systems making use of brochures or simple mobile applications, as patient-specific exercise prescriptions and exercise feedback are impossible. Alternatively, the interactive AR-based telerehabilitation used in this study may overcome the limitations of home exercise by providing comprehensive and easily accessible exercise instructions, thereby increasing compliance and enhancing exercise correctness through progress monitoring.

In conclusion, the RCT will provide evidence of “telerehabilitation” using hardware (IoT) and software (monitoring platform) technologies to develop “digital therapeutics” for the future.

### Trial status

The first participant recruitment began on 4 March 2020 and the study is expected to be completed at June, 2022.

## Supplementary Information

**Additional file 1.** SPIRIT Checklist.

**Additional file 2.** Exercises according to stages of adhesive capsulitis.

**Additional file 3.** Satisfaction questionnaire.

## Data Availability

Not applicable.

## Abbreviations

ROM: Range of motion
AR: Augmented reality
RCT: Randomized controlled trial
PROM: Passive range of motion
AROM: Active range of motion
ADL: Activities of daily living
NRS: Numerical rating scale
SPADI: Shoulder Pain and Disability Index
SF-36: 36-Item short Form Survey
EQ-5D-5L: European Quality of Life Five Dimensions Five Level Scale
