# Supplementary material for: Effectiveness of interactive augmented reality-based telerehabilitation in patients with adhesive capsulitis: protocol for a multi-center randomized controlled trial
Source: BMC Musculoskelet Disord. 2021 Apr 26;22:386. doi: 10.1186/s12891-021-04261-1 (PMC8074703; doi:10.1186/s12891-021-04261-1)
Supplement: Supplementary file 2 — Additional file 2. Exercises according to stages of adhesive capsulitis. [file 12891_2021_4261_MOESM2_ESM.docx]

**Additional file 1.** Exercises according to stages of adhesive capsulitis.

| **Freezing stage** | | | | | | | | | | | | | | | |
| --- | --- | --- | --- | --- | --- | --- | --- | --- | --- | --- | --- | --- | --- | --- | --- |
| **1-1. Warm up: Pendulum exercise** | | | | **1-2. Warm up: Abdominal breathing in the upright posture** | | | | | | | **2. Scapular stabilization: Scapular retraction exercise** | | | | |
| 1. Hold onto a chair or table with your unaffected arm and lean your upper body forward to allow the affected arm to hang in as relaxed a manner as possible. 2. Keep your trunk fixed and using only gravity as a weight, slowly rotate your arm clockwise and counterclockwise. 3. This exercise is even more effective with a light weight held in the hanging affected arm, i.e., a dumbbell or a 500-ml water bottle. 4. If you experience back pain during the exercise, straighten your back and rest for a while before proceeding. | | | | 1. Sit in a chair with a backrest, attach your back and buttocks to the backrest, and sit upright 2. Tilt your pelvis forward, straighten your back and chest, and keep your chin tilted up. Ensure that the waist and upper abdomen are not too far forward. 3. Relax your shoulders, place both hands on your stomach and inflate your stomach like a balloon while slowly inhaling through your nose. 4. Slowly exhale through your mouth while gently pushing your stomach in as if it were a balloon deflating. | | | | | | | 1. Raise your arms up to shoulder level, and then bend the elbows to a right angle. 2. Pull both arms back and bring both shoulder blades together. 3. Make sure you feel the muscles between your shoulder blades tighten. | | | | |
| **3. Shoulder range of motion exercise:** | | | | | | | | | | | | | | | |
| **Forward flexion (90°)** | **Abduction (90°)** | | | | | **External rotation (45°)** | | | | **Horizontal abduction and adduction** | | | **Extension** | | |
| 1. Sit in an upright position with a straight back and support the affected arm with the other. 2. Gradually raise the affected arm upward to the front of the body. 3. Stretch only until stiffness or just before you feel pain. | 1. Sit in an upright position with your back straight, hold opposite tips of the rod with both hands. 2. Gradually raise the affected arm upward to the side of the body. 3. Stretch only until stiffness or just before you feel pain. | | | | | 1. Bend your affected arm at a right angle and place a towel between your arm and body. 2. Holding the end of the rod with the affected arm and the middle with the other hand, gently push the rod outward. 3. Stretch only until stiffness or just before you feel pain. | | | | 1. Sit with your back straight. 2. Extend both arms forward and hold the rod, and then rotate in one direction at a time with open arms. 3. Stretch only until stiffness or just before you feel pain. | | | 1. Sit with your back straight. 2. Hold the rod with both hands behind your back and extend your arms, and then slowly lift upwards. 3. Try to feel an opening of the front shoulders and chest. 4. Stretch only until stiffness or just before you feel pain. | | |
| **5. Cool-down: Stretching** | | | | | | | | | | | | | | | |
| **Stretching 1: Chest stretch** | | | | **Stretching 2: Cross-body shoulder stretch** | | | | | | | **Stretching 3: Shoulder shrugs and roll** | | | | |
| 1. Stand upright and spread your legs shoulder-width apart, and then straighten your back. 2. Place the backs of your hands on your back, and then slowly raise your arms up. 3. Try to feel an opening of the front shoulders and chest. | | | | 1. Sit with your back straight. 2. Extend one arm forward and pull inward using the other arm. 3. Stretch and hold for as long as possible. | | | | | | | 1. Sit with your back straight. 2. Raise both shoulders toward your ears then roll them back. | | | | |
| **Frozen stage** | | | | | | | | | | | | | | |  |
| **1. Warm up: Abdominal breathing in the right posture** | | | | | | | **2. Scapular stabilization: Scapular retraction exercise** | | | | | | | |  |
| 1. Same as freezing stage | | | | | | | | | | | | | | |  |
| **3. Shoulder range of motion exercise:** | | | | | | | | | | | | | | |  |
| **Forward flexion** | | **Abduction** | | | **External rotation** | | | | **Horizontal abduction and adduction** | | | | | **Extension** |  |
| 1. Sit in an upright position with your back straight and support the affected arm with the other. 2. Gradually raise the affected arm upward to the front of the body. 3. **Go to the end and hold as long as possible.** | | 1. Sit in an upright position with your back straight, hold opposite tips of the rod with both hands. 2. Raise the affected arm, and slowly move the unaffected arm to the middle. 3. **Go to the end and hold as long as possible.** | | | 1. Bend your affected arm at a right angle and place a towel between your arm and body. 2. Holding the end of the rod with the affected arm and the middle with the other hand, gently push the rod outward. 3. **Go to the end and hold as long as possible.** | | | | 1. Sit with your back straight. 2. Extend both arms forward and hold the rod, and then rotate in one direction at a time with open arms. 3. **Go to the end and hold as long as possible.** | | | | | 1. Sit with your back straight. 2. Hold the rod with both hands behind your back and extend your arms, and then slowly lift upwards. 3. Try to feel an opening of the front shoulders and chest. 4. **Go to the end and hold as long as possible** |  |
| **4. Muscle strengthening with elastic band (yellow color)** | | | | | | | | | | | | | | |  |
| **Front raise** | | | **Lateral raise** | | | | | **Pull apart** | | | | **Single arm row** | | |  |
| 1. Sit with your back straight. 2. Fix one end of the rubber band under the sole of the foot, hold the other end and extend your arm forward, and then lift upwards. 3. Keep your trunk fixed, extend your arm, and lift forward. | | | 1. Sit with your back straight. 2. Fix one end of the rubber band to the leg of the chair, hold the other end, and lift your arm upwards to the side of your body. 3. With your trunk in the correct posture as shown in the picture, open your arms and raise it to the side. | | | | | 1. Sit with your back straight. 2. Bend your arms at right angles, hold the rubber band at both ends, and open your forearms to the sides. 3. Keep your arms open then slowly return to their original place. | | | | 1. Sit with your back straight. 2. Fold the rubber band in half, fix the band under the sole of the foot, and hold both ends in one hand. 3. Pull the band back while bending your elbow. | | |  |
| **5. Cool-down: Stretching** | | | | | | | | | | | | | | |  |
| **Stretching 1: Chest stretch** | | | **Stretching 2: Shoulder pull (Triceps stretch)** | | | | | **Stretching 3: Cross-body shoulder stretch** | | | | **Stretching 4: Shoulder shrugs and roll** | | |  |
| 1. Sit with your back straight. 2. Place both hands on the back of the head with elbows facing each other, insert the pods, and then open and close both elbows. 3. Stretch and hold for as long as possible. | | | 1. Sit with your back straight. 2. Bend one arm over and behind your head, and then pull your elbow toward the middle with the other hand. 3. Stretch and hold for as long as possible. | | | | | 1. Sit with your back straight. 2. Extend one arm forward and pull inward using the other arm. 3. Stretch and hold for as long as possible. | | | | 1. Sit with your back straight. 2. Raise both shoulders toward your ears then roll them back. | | |  |

| **Thwaing stage** | | | | | | | | |
| --- | --- | --- | --- | --- | --- | --- | --- | --- |
| **1. Warm up: Abdominal breathing in the right posture** | | | | **2. Scapular stabilization: Scapular retraction exercise** | | | | |
| 1. Same as freezing stage | | | | | | | | |
| **3. Shoulder range of motion exercise:** | | | | | | | | |
| **Forward flexion** | **Abduction** | | **External rotation** | | | **Horizontal abduction and adduction** | | **Extension** |
| 1. Sit in an upright position with your back straight and support the affected arm with the other. 2. Gradually raise the affected arm upward to the front of the body. 3. **Go to the end and hold as long as possible.** | 1. Sit in an upright position with your back straight; hold opposite tips of the rod with both hands. 2. Raise the affected arm, and slowly move the unaffected arm to the middle. 3. **Go to the end and hold as long as possible.** | | 1. Bend your affected arm at a right angle and place a towel between your arm and body. 2. Holding the end of the rod with the affected arm and the middle with the other hand, gently push the rod outward. 3. **Go to the end and hold as long as possible.** | | | 1. Sit with your back straight. 2. Extend both arms forward and hold the rod, and then rotate in one direction at a time with open arms. 3. **Go to the end and hold as long as possible.** | | 1. Sit with your back straight. 2. Hold the rod in both hands behind your back and extend your arms, and then slowly lift upwards. 3. Try to feel an opening of the front shoulders and chest. 4. **Go to the end and hold as long as possible** |
| **4. Muscle strengthening with dumbbell (0.5 kg)** | | | | | | | | |
| **Seated External rotation** | **Biceps curl** | | **Scaption** | | | **Shoulder press** | | **Elbow extension** |
| 1. Sit sideways on a chair with your back straight, lift a dumbbell in one hand, bend your arm and place your elbow on the backrest of the chair. 2. Keep your arm bent and raise your forearm upward. 3. Slowly lower your forearms on the back of the chair. | 1. Sit in an upright position with your back straight; lift dumbbells in both hands. 2. Slowly bend both arms toward you. 3. After lifting your arms, slowly lower them to the side of your trunk. | | 1. Sit in an upright position and diagonally lift dumbbells outward to the height of your shoulders, with your thumbs facing the ceiling. 2. Slowly lower your elbows. | | | 1. Sit in an upright position with your back straight; lift dumbbells with both hands. 2. Lift your arms to the side of your body at a right angle. Hold the dumbbell with your palms facing the front. 3. Extend your elbows straight up, and then slowly lower them down. | | 1. Sit in an upright position with your back straight and lift a dumbbell with one hand. 2. Bend it over the back of your head and straighten your elbow to lift the dumbbell over your head, and then slowly back down. 3. If necessary, assist the elbow with the other hand. |
| **5. Cool-down: Stretching** | | | | | | | | |
| **Stretching 1: Chest stretch** | | **Stretching 2: Shoulder pull (Triceps stretch)** | | | **Stretching 3: Cross-body shoulder stretch** | | **Stretching 4: Shoulder shrugs and roll** | |
| 1. Sit with your back straight. 2. Place both hands on the back of the head with elbows facing each other, insert the pods, and then open and close both elbows. 3. Stretch and hold for as long as possible. | | 1. Sit with your back straight. 2. Bend one arm over and behind your head, and then pull your elbow toward the middle with the other hand. 3. Stretch and hold for as long as possible. | | | 1. Sit with your back straight. 2. Extend one arm forward and pull inward using the other arm. 3. Stretch and hold for as long as possible. | | 1. Sit with your back straight. 2. Raise both shoulders toward your ears then roll them back. | |
