# Supplementary material for: Effectiveness of interactive augmented reality-based telerehabilitation in patients with adhesive capsulitis: protocol for a multi-center randomized controlled trial
Source: BMC Musculoskelet Disord. 2021 Apr 26;22:386. doi: 10.1186/s12891-021-04261-1 (PMC8074703; doi:10.1186/s12891-021-04261-1)
Supplement: Supplementary file 3 — Additional file 3. Satisfaction questionnaire. [file 12891_2021_4261_MOESM3_ESM.docx]

Appendix 2

Satisfaction Questionnaire

Q1. What was the quality of the service you received?

1. Unsatisfied
2. Neutral
3. Satisfied
4. Very Satisfied

Q2. Did you get the services you wanted?

1. Not at all
2. No
3. Yes
4. Yes, I got very wanted service

Q3. To what extent have the services you participated in met your needs?

1. Not at all
2. Somewhat met
3. Mostly met
4. All met

Q4. How satisfied were you with the services you received?

1. Very unsatisfied
2. Somewhat unsatisfied
3. Somewhat Satisfied
4. Very Satisfied

Q5. If someone you know is in a similar situation, would you recommend participating in this program?

1. Never
2. No
3. Yes
4. Yes, actively recommend

Q6. Did the services you get help you effectively solve your problem?

1. Not at all
2. No
3. Yes, somewhat helpful
4. Yes, very helpful

Q7. How satisfied are you with the program and services overall?

1. Very unsatisfied
2. Unsatisfied
3. Satisfied
4. Very satisfied

Q8. Would you rejoin this program if you need these services again in the future?

1. Never
2. No
3. Yes
4. Yes, I will actively rejoin

Q9. Please write down any inconveniences in using the service or items that need to be improved

________________________________________________________________

________________________________________________________________

________________________________________________________________

________________________________________________________________

________________________________________________________________

Q10. Please write down what you liked while using the service

________________________________________________________________

________________________________________________________________

________________________________________________________________

________________________________________________________________

________________________________________________________________
